# An in situ approach to characterizing photosynthetic gas exchange of rice panicle

**DOI:** 10.1186/s13007-020-00633-1

**Published:** 2020-07-06

**Authors:** Tian-Gen Chang, Qing-Feng Song, Hong-Long Zhao, Shuoqi Chang, Changpeng Xin, Mingnan Qu, Xin-Guang Zhu

**Affiliations:** 1 National Key Laboratory for Plant Molecular Genetics, Center of Excellence for Molecular Plant Sciences, Chinese Academy of Sciences, Shanghai, 200031 China; 2 University of Chinese Academy of Sciences, Beijing, 100049 China; 3 State Key Laboratory of Hybrid Rice, Hunan Hybrid Rice Research Center, Changsha, China; 4 CAS Key Laboratory for Computational Biology, CAS-MPG Partner Institute for Computational Biology, Shanghai Institute of Nutrition and Health, Chinese Academy of Sciences, Shanghai, 200031 China

**Keywords:** Rice panicle photosynthesis, Non-foliar photosynthesis, Reproductive organs, In situ photosynthetic characteristics phenotyping, Grain setting rate, P-chamber

## Abstract

**Background:**

Photosynthesis of reproductive organs in C_3_ cereals is generally regarded as important to crop yield. Whereas, photosynthetic characteristics of reproductive organs are much less understood as compared to leaf photosynthesis, mainly due to methodological limitations. To date, many indirect methods have been developed to study photosynthesis of reproductive organs and its contribution to grain yield, such as organ shading, application of herbicides and photosynthetic measurement of excised organs or tissues, which might be intrusive and cause biases. Thus, a robust and in situ approach needs to be developed.

**Results:**

Here we report the development of a custom-built panicle photosynthesis chamber (P-chamber), which can be connected to standard infrared gas analyzers to study photosynthetic/respiratory rate of a rice panicle. With the P-chamber, we measured panicle photosynthetic characteristics of seven high-yielding elite *japonica*, *japonica*-*indica* hybrid and *indica* rice cultivars. Results show that, (1) rice panicle is photosynthetically active during grain filling, and there are substantial inter-cultivar variations in panicle photosynthetic and respiratory rates, no matter on a whole panicle basis, on an area basis or on a single spikelet basis; (2) among the seven testing cultivars, whole-panicle gross photosynthetic rates are 17–54 nmol s^−1^ 5 days after heading under photon flux density (PFD) of 2000 μmol (photons) m^−2^ s^−1^, which represent some 20–38% of that of the corresponding flag leaves; (3) rice panicle photosynthesis has higher apparent CO_2_ compensation point, light compensation point and apparent CO_2_ saturation point, as compared to that of a typical leaf; (4) there is a strong and significant positive correlation between gross photosynthetic rate 5 days after heading on a single spikelet basis and grain setting rate at harvest (Pearson correlation coefficient r = 0.93, *p* value < 0.0001).

**Conclusions:**

Rice panicle gross photosynthesis is significant, has great natural variation, and plays an underappreciated role in grain yield formation. The P-Chamber can be used as a tool to study in situ photosynthetic characteristics of irregular non-foliar plant organs, such as ears, culms, leaf sheaths, fruits and branches, which is a relatively less explored area in current cereal breeding community.

## Background

In cereal crops such as rice and wheat, there are two major sources of assimilates used for grain filling, i.e., the post-anthesis photosynthesis and the pre-anthesis assimilates stored in the stem. Under non-stress conditions, the post-anthesis photosynthesis contributes greater to grain yield than the pre-anthesis stored assimilates [1–4]. Leaves, especially flag leaves located at the upmost of stems, have been believed to be the dominant contributor of post-anthesis photosynthesis [5, 6]. Nevertheless, reproductive organs, including glumes, caryopsis pericarps, and awns, are green before mature and photosynthetically active [7, 8], suggesting a possibility of them being a major source of photosynthate for grain filling as well. In fact, reproductive organs have several potential advantages over leaves in terms of photosynthate production and transportation: (1) they generally locate at the upper canopy, where light levels are higher than those at the lower canopy; (2) they are physically closer to the developing grains than the leaves, which shorten the path of assimilates transport to developing grains [9]; (3) they have large surface area, e.g. the ear surface areas were 1.1-5.5 folds greater than that of the corresponding flag leaves in wheat and barley [6]; 4) reproductive organs tend to senesce later than leaves, especially under adverse conditions such as drought [10, 11]. Due to these reasons, photosynthesis of reproductive organs gained more and more attraction in recent years (see reviews in [12–15]).

In wheat and barley, ear photosynthesis is a major source of photosynthate for grain filling; a large photosynthetic area and high photosynthetic activity from both the green glumes and awns are major factors underlying higher ear photosynthesis [6, 16, 17]. Using ear-shading and/or stem-shading methods, ear photosynthesis was estimated to contribute 10% to 65% of the grain yield in different wheat lines and under different growth conditions [16–20]. By analyzing the carbon isotope composition in different organs, it was estimated that ear photosynthesis contributed 47% to 100% of the grain yield in six high-yielding bread wheat lines under well-managed conditions [21, 22]. In a retrospective study, by using 15 winter wheat cultivars released from 1980 to 2012 in North China Plain, it was reported that the contribution of ear photosynthesis to grain yield doubled (from around 30% to around 60%) during breeding [18]. Although the exact proportion of ear photosynthesis to total grain yield varies, breeding cultivars with higher ear photosynthesis was recognized as a new mechanism to improve wheat yield potential [14, 15, 23].

In contrast to the significant importance of ear photosynthesis in wheat and barley, rice panicle has been traditionally regarded as a sink organ with negligible photosynthetic contribution to grain yield [24]. For example, Tsuno et al. (1975) estimated that net panicle photosynthesis is about 5% of that of an active photosynthesizing rice leaf [25]; Takeda and Maruta (1956) estimated that panicle photosynthesis contributes 8% of the rice grain yield [26]. Reflecting the notion of rice ear photosynthesis being of little importance to grain filling, some researchers recommended lowering spike position in a canopy as much as possible to increase canopy photosynthesis by minimizing shading leaves by panicles [27].

Is panicle photosynthesis in rice really of little importance for grain filling? More and more evidences are suggesting the opposite. For example, Imaizumi et al. (1990) found that whole-panicle gross amount of photosynthetically assimilated CO_2_ was 30% of that in a flag leaf, and spikelets had a similar photosynthetic capability to a flag leaf on the basis of Chl [8]. It has been shown that rice panicle contains higher amount of chloroplast NAD(P)H dehydrogenase-like complex, and maintains a similar or even higher quantum yield of PSII than that of the flag leaf from heading to harvest [28]. There are also evidences showing a potential important role of rice panicle photosynthesis in maintaining grain filling under moderate stress conditions. For example, under moderate wetting and drying irrigation after anthesis, genes that function in photosynthesis and carbohydrate metabolism were upregulated in inferior spikelets, together with a simultaneous increase of grain filling rate and grain weight of the inferior spikelets [29].

One major factor attributing to the inconsistency regarding the importance of photosynthesis of reproductive organs in grain filling is to the lack of robust methods to measure photosynthesis in organs with a complex three-dimensional geometry [12, 22, 23]. Many indirect methods have been developed to study photosynthesis of reproductive organs [23], e.g. through ear (awn) removal/shading or application of herbicides [17, 27, 30], isotope tracing [21], gas exchange measurement of excised spikelets [6, 8] and chlorophyll fluorescence measurement for plants cultured in pots [28]. Among these methods, the shading treatments or applications of herbicides may affect other function of treated organs and/or trigger compensation effects of unaffected organs [20, 22, 31–35]. The stable isotope discrimination method calculated contributions of different organs to grain yield based on instant carbon isotope in water-soluble fraction in different tissues at mid-grain filling stage and carbon isotope in mature grains [12, 21, 22]. The accuracy of this isotope discrimination method might be affected by the dynamic environment (e.g. temperature and air humidity) and physiological processes other than photosynthesis (e.g. assimilates transport and ear night respiration) during the whole grain filling season [12, 21, 22]. Gas exchange measurement on excised ears or spikelets is an intrusive method and it can cause bias due to an alteration of ear natural architecture and posture [27].

Thus, the main objectives of this study are: (1) to develop a direct and non-intrusive method for gas exchange measurement of rice panicles; (2) to explore panicle photosynthetic characteristics and the inter-cultivar variations of photosynthetic gas exchange parameters; (3) and to study the relationship between panicle photosynthetic gas exchange parameters and grain yield related agronomic traits.

## Results

### Performance of the panicle photosynthesis P-chamber

**Fig. 1:**
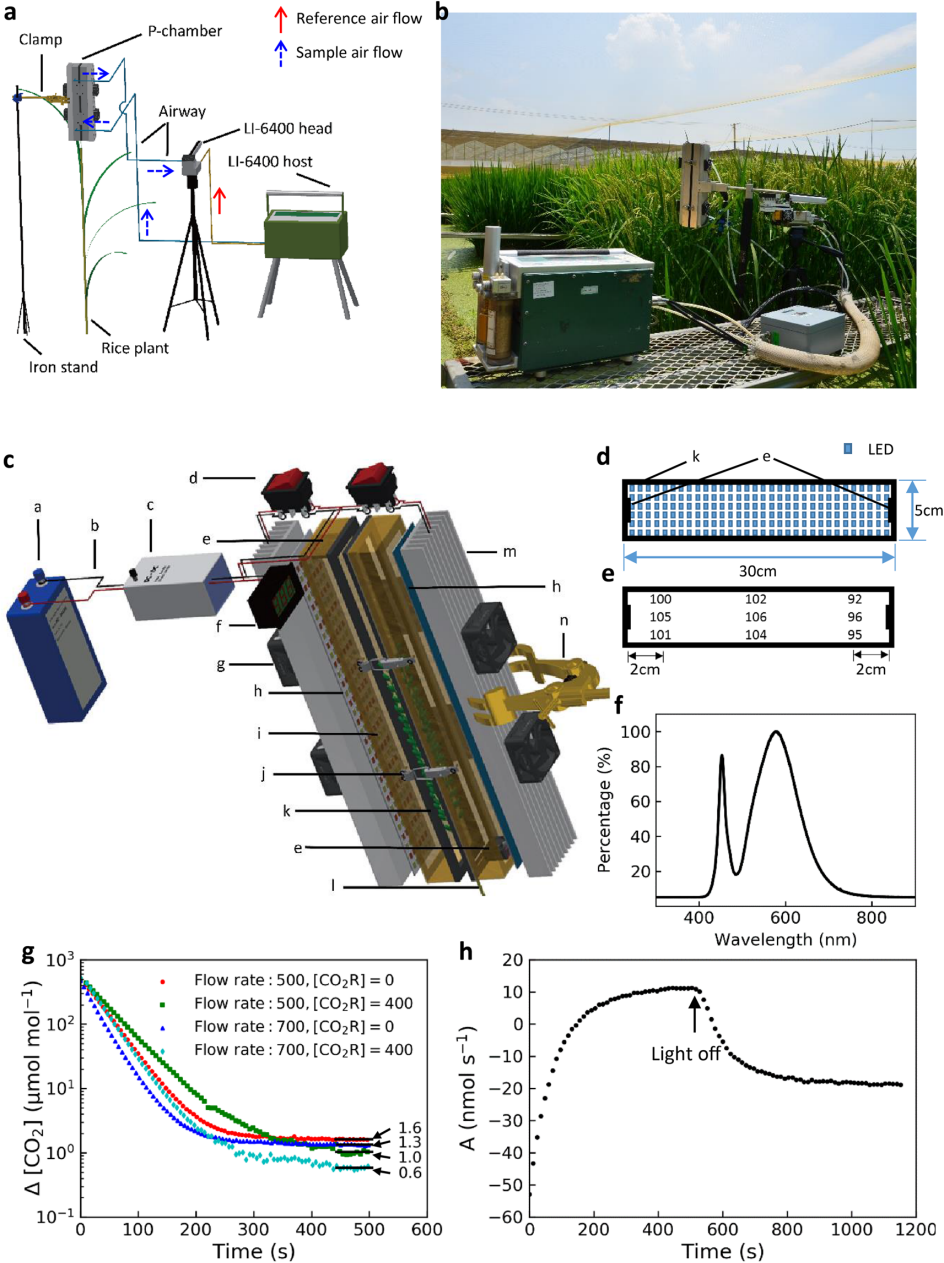
A custom-built panicle photosynthesis chamber (P-chamber) for panicle gas exchange measurements. **a** An illustration of how the P-chamber works together with the gas analyzer Li-6400 to measure the photosynthetic and respiratory rates of a rice panicle. **b** The setting of a P-chamber in a paddy field. **c** An illustration of design of the P-chamber. **d** A top-down view of the inner part of the P-chamber. **e** The homogeneity of PFD in the chamber. **f** Spectrum of the LED light source. **g** The difference between the CO_2_ concentration in the P-chamber and the set CO_2_ concentration ([CO_2_R]) in the reference pipe at different time after closure of the P-chamber. **h** Recorded photosynthetic gas exchange rate of a rice panicle under a photon flux density (PFD) of 2000 μmol (photons) m^−2^ s^−1^ followed by dark treatment. Letters in panel (**a**) and (**b**): **a** Battery; **b** Wires; **c** Voltage adaptor; **d** Switch; **e** Mixing fan (inside the chamber body); **f** Digital displayed voltmeter; **g** Cooling fan; **h** LED array; **i** Chamber body; **j** Hasp; **k** Sealing strip; **l** Rice panicle; **m** Cooling fin; **n** Clamp. The horizontal lines in panel (**g**) represent mean values of the last 10 data points. The y-axis in panel (**g**) is log-scaled

The P-chamber is connected to a gas analyzer Li-6400 (Li-Cor Inc., Lincoln, Nebraska, USA) to measure gas exchange rate of a rice panicle (Fig. 1a, b). The panicle is illuminated with LED light source (Fig. 1c, d). The LED light source emits pure white light (light wavelength information is shown in Fig. 1f). The photon flux density (PFD) is in a range of ± 10% of set value on 87% of the total 30 × 5 cm light field (Fig. 1e). At a room temperature of 25 °C with a flow rate of 700 μmol s^−1^, temperature rise in the chamber at PFD of 2000 μmol (photons) m^−2^ s^−1^ is 4.3 ± 0.7 °C.

To test the leakage of the P-chamber, we recorded Δ[CO_2_] (the difference between CO_2_ concentration in the P-chamber and the set reference CO_2_ concentration in the reference pipe) at different time after closure of the P-chamber. With an initial Δ[CO_2_] value of around 500 μmol mol^−1^, the Δ[CO_2_] values were stabilized within 8 min under different flow rates and different CO_2_ concentrations (Fig. 1g). The final Δ[CO_2_] values were ≤ 1.6 μmol mol^−1^ and ≤ 1.3 μmol mol^−1^ at a flow rate of 500 and 700 μmol s^−1^, respectively (Fig. 1g). During a typical measurement of panicle photosynthetic rate and respiratory rate, the light source was turned on before the rice panicle was enclosed, and the photosynthetic rate was recorded after photosynthetic rate reading was stabilized; then the light source was turned off, and the respiratory rate was recorded after respiratory rate reading was stabilized (Fig. 1h). A comprehensive measurement for photosynthetic and respiratory rates of a rice panicle usually took 10 ~ 20 min after the panicle was enclosed in the P-chamber, depended on size and photosynthetic activity of the panicle.

### Quantification of rice panicle photosynthesis

We first used the P-chamber to measure the in situ whole-panicle photosynthetic gas exchange parameters 5 days after heading for different rice cultivars when the panicles were still erect or semi-erect. Specifically, whole-panicle net photosynthetic rate (panicle *A*_net_) and dark respiratory rate (panicle *R*_d_) were measured; and whole-panicle gross photosynthetic rate (panicle *A*_gross_) was calculated as a sum of panicle *A*_net_ and panicle *R*_d_. In addition, average spikelet *A*_gross_, *A*_net_ and *R*_d_ were calculated by dividing panicle *A*_gross_, *A*_net_ and *R*_d_ by the spikelet number of a panicle.

**Fig. 2:**
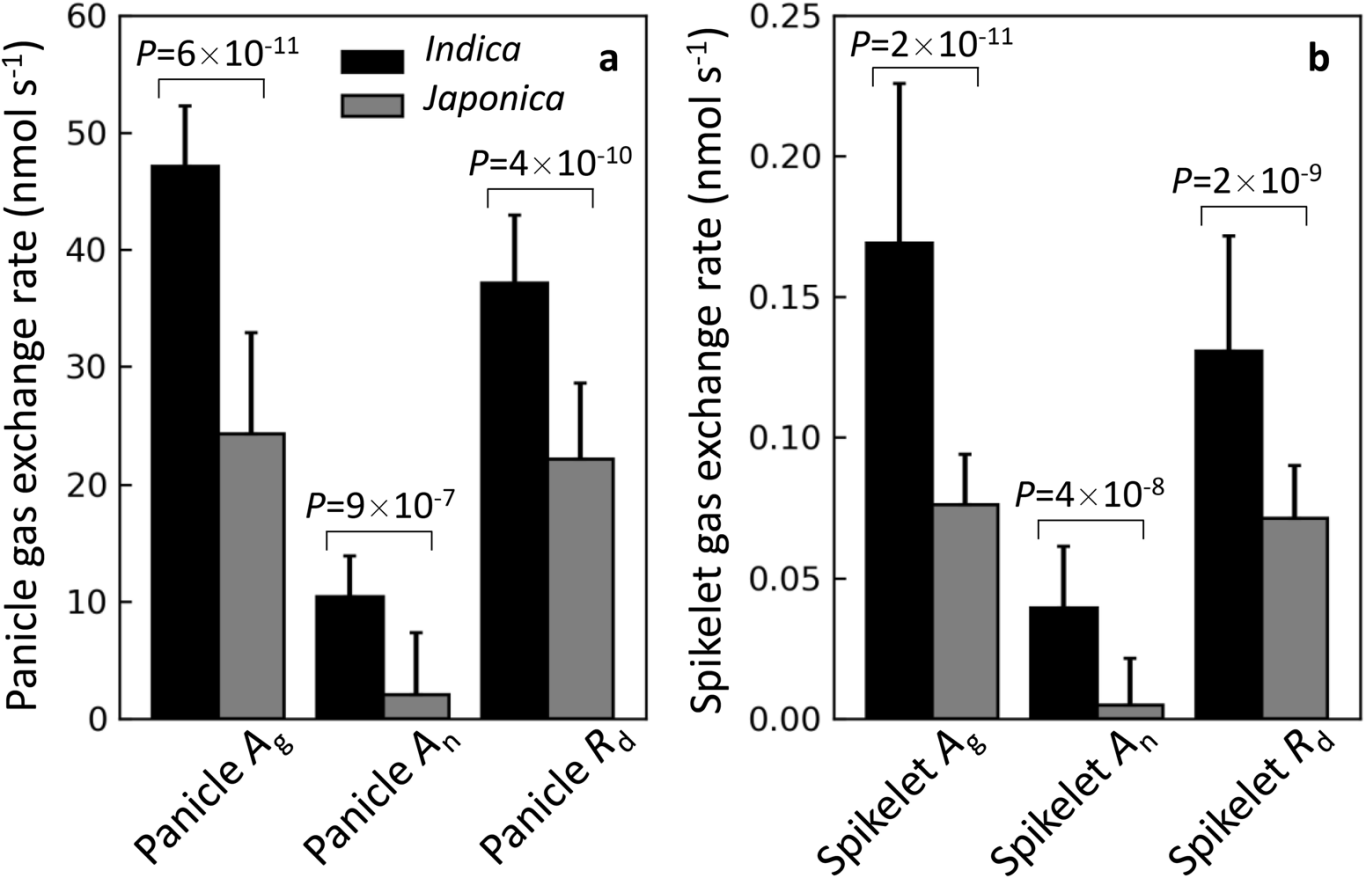
Comparison of the gas exchange rates between indica and japonica rice cultivars 5 days after heading. **a** Gross photosynthetic, net photosynthetic and respiratory rates of a whole rice panicle. **b** Average gross photosynthetic, net photosynthetic and respiratory rates of a spikelet. Data of indica group were combined from rice cultivars YLY900, CY1000, SY63 and 9311; Data of japonica group were combined from rice cultivars XS134, YY538 and YY17. Data were combined from 2015 and 2016 experiments. Statistically different p-values were calculated from Student’s t-test

Notably, substantial inter-cultivar variations in photosynthetic gas exchange parameters among rice cultivars were found, for both values of a whole panicle and values normalized by spikelet number. For example, the spikelet *A*_net_ and panicle *A*_net_ were 0.012 ~ 0.051 nmol s^−1^ and 2.7 ~ 12.0 nmol s^−1^ in 2015, and were −0.014 ~ 0.075 nmol s^−1^ and −3.3 ~ 15.0 nmol s^−1^ in 2016, respectively (Table 1). Panicle *A*_gross_ varied from 17.1 to 53.6 nmol s^−1^ under PFD of 2000 μmol (photons) m^−2^ s^−1^ for the seven rice cultivars (Table 1). Moreover, *indica* type rice cultivars (YLY900, CY1000, SY63 and 9311) had an overall significantly higher *A*_gross_, *A*_net_ and *R*_d_ than *japonica* and *japonica*-*indica* hybrid type rice cultivars (XS134, YY538 and YY17), both on whole panicle basis and on single spikelet basis (Fig. 2).

**Table 1:** Panicle photosynthetic gas exchange parameters of the rice cultivars measured 5 days after heading

Year	Cultivar	Panicle *A*_net_ (nmol s^−1^)	Panicle *A*_gross_ (nmol s^−1^)	Panicle *R*_d_ (nmol s^−1^)	Spikelet *A*_net_ (nmol s^−1^)	Spikelet *A*_gross_ (nmol s^−1^)	Spikelet *R*_d_ (nmol s^−1^)
2015	YLY900	12 ± 2.4a	49 ± 2.8a	37 ± 1.5a	0.035 ± 0.003a	0.147 ± 0.011b	0.111 ± 0.013a
	CY1000	7.2 ± 1.3b	43.6 ± 7a	36.4 ± 7.3a	0.015 ± 0.004b	0.091 ± 0.005c	0.075 ± 0.005b
	SY63	11.8 ± 2.2a	42.8 ± 2.7a	31 ± 2.8a	0.051 ± 0.012a	0.185 ± 0.014a	0.133 ± 0.007a
	9311	10.9 ± 1.4ab	46 ± 3.4a	35 ± 3.9a	0.044 ± 0.009a	0.182 ± 0.007ab	0.138 ± 0.003a
	XS134	2.7 ± 1.1c	18.1 ± 3.4b	15.4 ± 2.5b	0.012 ± 0.005b	0.078 ± 0.022c	0.067 ± 0.017b
2016	YLY900	7.3 ± 2.4b	47.5 ± 4.1a	40.2 ± 5.6a	0.021 ± 0.007bc	0.136 ± 0.011b	0.115 ± 0.014b
	CY1000	6.9 ± 2.2b	44.3 ± 4ab	37.4 ± 4.6ab	0.017 ± 0.006bc	0.106 ± 0.013bc	0.089 ± 0.01bc
	SY63	15 ± 3a	49.6 ± 4.3a	34.6 ± 5.3ab	0.075 ± 0.016a	0.248 ± 0.021a	0.172 ± 0.023a
	9311	11.9 ± 2.6ab	53.6 ± 3.9a	45.5 ± 4.5a	0.052 ± 0.009a	0.243 ± 0.005a	0.205 ± 0.021a
	XS134	−3.3 ± 3.4c	17.1 ± 4.4d	20.3 ± 6.4c	−0.014 ± 0.014d	0.075 ± 0.018 cd	0.089 ± 0.026bc
	YY538	−0.4 ± 2.4c	25.8 ± 4.7 cd	26.2 ± 4.1bc	−0.001 ± 0.006 cd	0.06 ± 0.009d	0.062 ± 0.011c
	YY17	9.3 ± 3.1ab	36 ± 5.8bc	26.8 ± 5.6bc	0.024 ± 0.009b	0.091 ± 0.01 cd	0.068 ± 0.01c

Data presented are mean values with s.d. (n = 3 in year 2015; n = 5 in year 2016). Data followed by different letters are statistically different at *p*-value < 0.05

In 2016, we further measured area of panicles and area of the corresponding flag leaves, and then quantified *A*_gross_, *A*_net_ and *R*_d_ on an area basis by dividing panicle *A*_gross_, *A*_net_ and *R*_d_ by the area of a panicle (Additional file 1: Figure S1; see detailed procedure used to calculate panicle surface area in Materials and Methods). As a result, we found spikelets contributed the majority of the panicle area, whereas branches only accounted for 24 ~ 33% of the panicle area; moreover, total area of a panicle (single side) was 72 ~ 121 cm^2^, which was 20 ~ 124% higher than that of a flag leaf (Table 2). On an area basis, *A*_net_ of panicles were very low, i.e., only − 0.6 ~ 2.2 μmol m^−2^ s^−1^, under a PFD of 2000 μmol (photons) m^−2^ s^−1^; whereas *A*_gross_ were 2.1 ~ 7.2 μmol m^−2^ s^−1^, much higher than *A*_net_, as a result of high *R*_d_, i.e., 2.1 ~ 6.1 μmol m^−2^ s^−1^ (Table 3).

**Table 2:** The area of a flag leaf and the areas of all spikelets and branches in a panicle for different rice cultivars 5 days after heading (n = 5)

Name	Spikelets area (cm^2^)	Branches area (cm^2^)	Panicle area (cm^2^)	Branches: Panicle area ratio	Flag leaf area (cm^2^)
YLY900	79.7 ± 6.5a	34.6 ± 2.8a	115.5 ± 11.7ab	0.3 ± 0.01b	73.8 ± 11b
CY1000	74.7 ± 7.9a	28.4 ± 1.4b	120.7 ± 13a	0.28 ± 0.02 cd	88.1 ± 11.3a
SY63	47 ± 4.1bc	23 ± 1.9 cd	74.3 ± 7.2d	0.33 ± 0a	61.9 ± 10.9 cd
9311	54.2 ± 4.2b	21.2 ± 1.6d	71.6 ± 6.8d	0.28 ± 0bc	56.2 ± 12.1de
XS134	39 ± 3.5c	12.6 ± 1.4e	56.3 ± 8.1e	0.24 ± 0.03d	32.6 ± 4.4f
YY538	69.5 ± 3.7a	26.8 ± 1.6bc	109 ± 9.1bc	0.28 ± 0.02bcd	48.7 ± 8e
YY17	72.5 ± 6.7a	25.6 ± 3.1bcd	106.3 ± 8.7c	0.26 ± 0.01 cd	72.3 ± 8.4bc

Data measured in 2016. Data presented are mean values with s.d. (n = 5). Data followed by different letters are statistically different at *p*-value < 0.05. Note that areas presented are single side based

**Table 3:** Panicle photosynthetic gas exchange parameters on an area basis for different rice cultivars 5 days after heading (n = 5)

Name	*A*_net_ per unit area (μmol m^−2^ s^−1^)	*A*_gross_ per unit area (μmol m^−2^ s^−1^)	*R*_d_ per unit area (μmol m^−2^ s^−1^)
YLY900	0.7 ± 0.2 cd	4.2 ± 0.4b	3.6 ± 0.4bc
CY1000	0.6 ± 0.2 cd	3.6 ± 0.4bc	3 ± 0.3bcd
SY63	2.2 ± 0.5a	7.2 ± 0.6a	5 ± 0.7ab
9311	1.6 ± 0.3ab	7.2 ± 0.1a	6.1 ± 0.6a
XS134	−0.6 ± 0.6e	3 ± 0.7 cd	3.6 ± 1.1bc
YY538	0 ± 0.2de	2.1 ± 0.3d	2.1 ± 0.4d
YY17	0.9 ± 0.3bc	3.5 ± 0.4bc	2.6 ± 0.4 cd

Data were measured in 2016. Data presented are mean values with s.d. (n = 5). Data followed by different letters are statistically different at *p*-value < 0.05

**Fig. 3:**
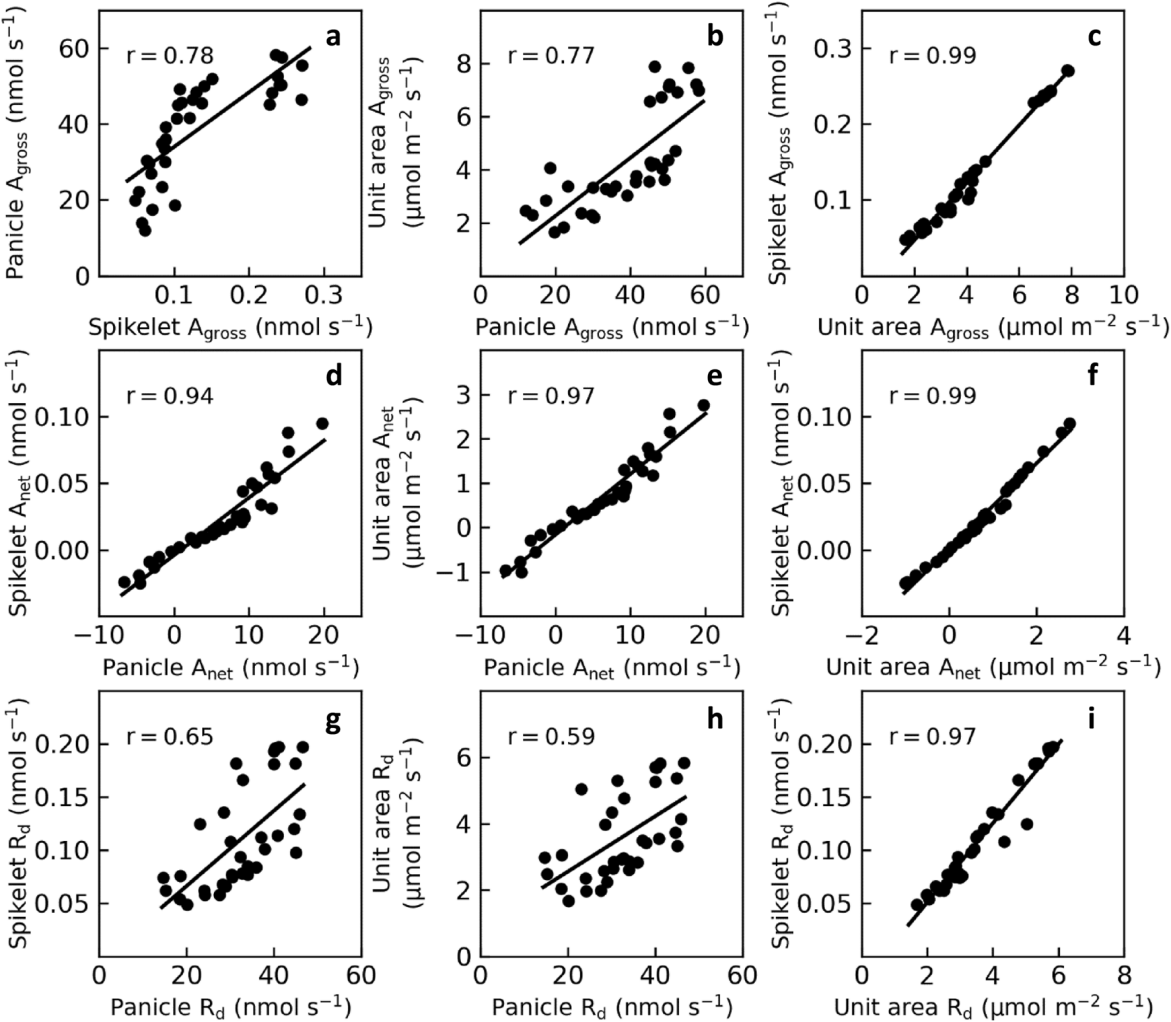
Correlations between panicle photosynthetic gas exchange parameters normalized on a panicle basis, on a spikelet basis and on an area basis. **a–c** Correlations between *A*_gross_ of panicles normalized on different bases. **d–f** Correlations between *A*_net_ of panicles normalized on different bases. **g–i** Correlations between *R*_d_ of panicles normalized on different bases. Data measured in 2016 (n = 35)

**Fig. 4:**
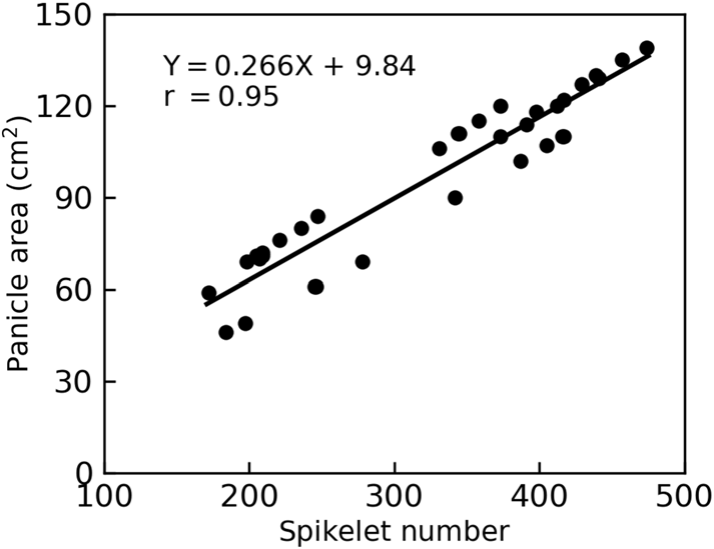
Correlation between panicle area and spikelet number. Data measured in 2016 (n = 35)

Given these different representations of gas exchange rates, i.e., on a panicle basis, on a spikelet basis and on an area basis, what’s the relations between them? Positive correlations between values on different bases were found for all the three parameters *A*_gross_, *A*_net_ and *R*_d_ (the Pearson correlation coefficient r = 0.59 ~ 0.99; Fig. 3a–i). Interestingly, we found that *A*_net_ per panicle was strongly positively correlated with *A*_net_ per unit panicle area (the Pearson correlation coefficient r = 0.97; Fig. 3e), in contrast to that for *A*_gross_ and *R*_d_ (the Pearson correlation coefficient r = 0.77 and 0.59, respectively; Fig. 3b, h). In addition, spikelet based *A*_gross_, *A*_net_ and *R*_d_ were closely related to area based *A*_gross_, *A*_net_ and *R*_d_ (the Pearson correlation coefficient r = 0.97–0.99; Fig. 3c, f, i), as a result of a tight relationship between panicle area and spikelet number (the Pearson correlation coefficient r = 0.95; Fig. 4).

### Characterization of rice panicle photosynthesis

**Fig. 5:**
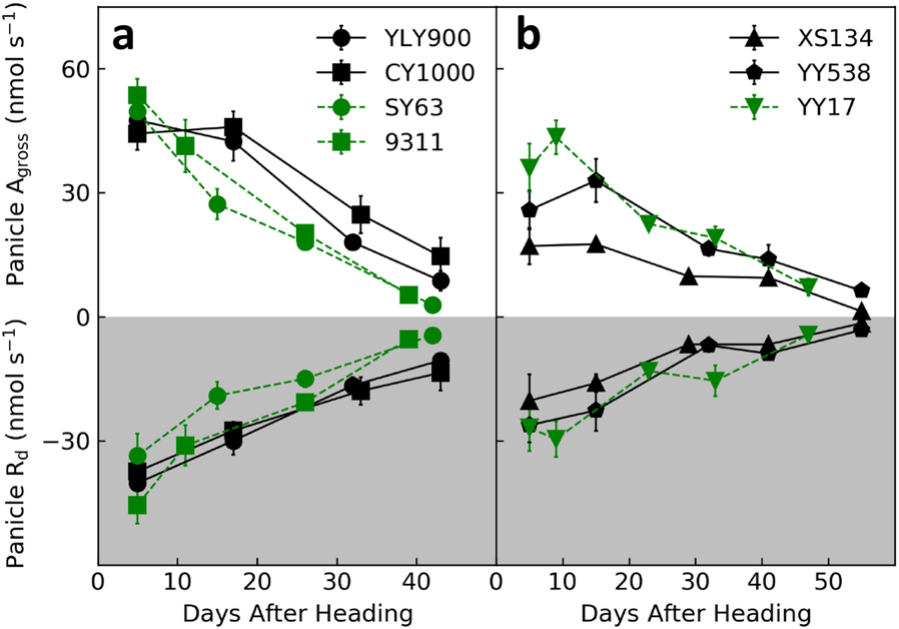
The dynamic changes of panicle gross photosynthetic and respiratory rates during grain filling for seven rice cultivars. Panel (**a)** and (**b)** illustrate the four *indica* cultivars and the three *japonica* or *japonica*-*indica* hybrid cultivars, respectively. Data measured in 2016. Data presented are mean values with s.d. (n = 5)

To further characterize rice panicle photosynthesis, we measured dynamic changes of panicle *A*_gross_ and *R*_d_ for seven rice cultivars during grain filling in 2016 (Fig. 5). Firstly, panicle *A*_gross_ and *R*_d_ were highest at early grain filling stage, then slowly decreased over time. Notably, both *A*_gross_ and *R*_d_ could still be detected even 40 days after heading, i.e., panicle photosynthesis and respiration were active throughout the grain-filling season. It is worth mentioning here that the panicle *A*_gross_ should be interpreted as the maximal photosynthetic potential, as the panicle was held upright during measurement, which was not the natural state after early grain filling stage for some cultivars.

**Fig. 6:**
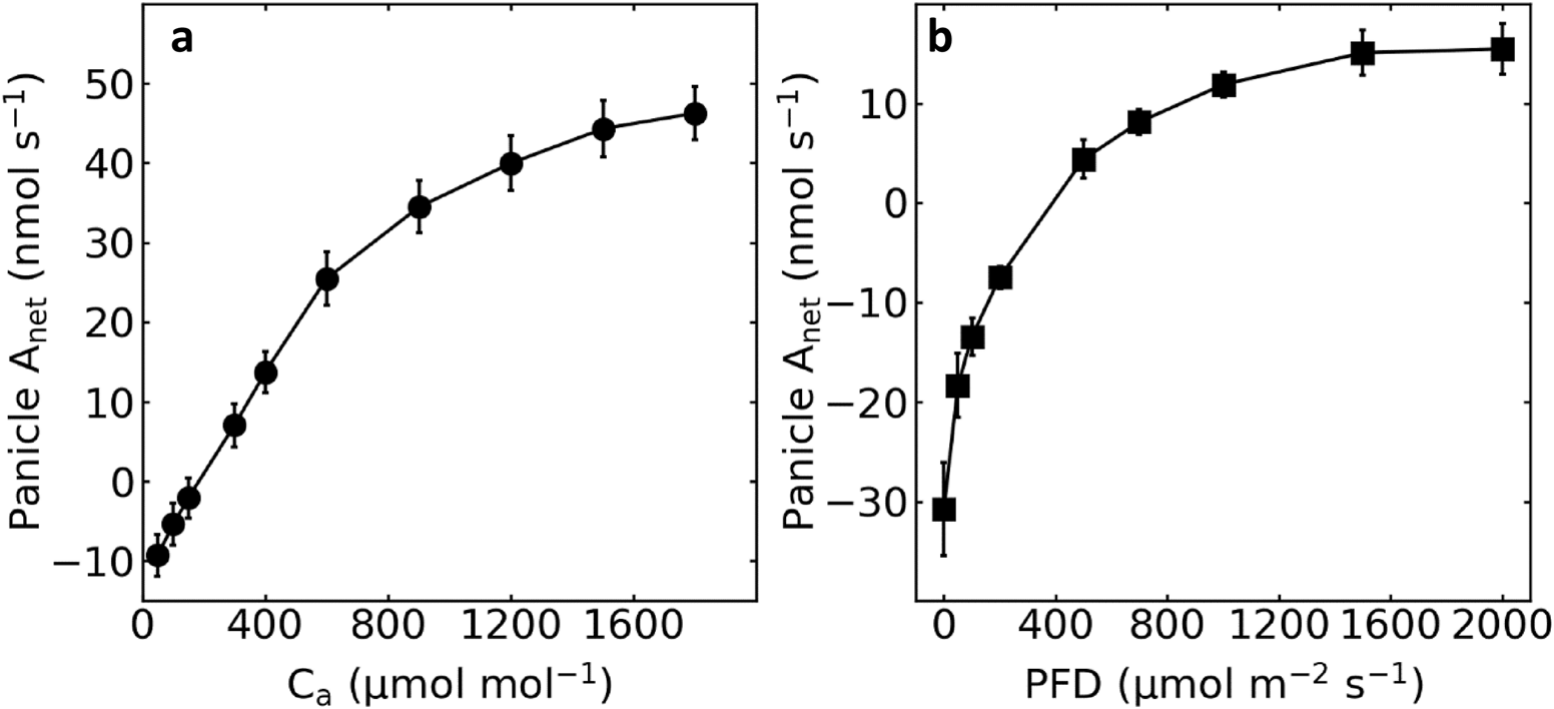
Panicle photosynthetic CO_2_ response curve (**a**) and light response curve (**b**) of rice cultivar YY17. Data measured 10 days after heading in 2016. Data presented are mean values with s.d. (n = 3)

We further measured the photosynthetic light and CO_2_ response patterns of a panicle using rice cultivar YY17. Although the shapes of both response curves are similar to that of a leaf, there are several differences. Firstly, the apparent CO_2_ and light compensation points of a panicle were about 180 μmol mol^−1^ and 390 μmol m^−2^ s^−1^ (Fig. 6), much higher than those of a leaf, which are usually < 100 μmol mol^−1^ and < 50 μmol m^−2^ s^−1^, respectively. Secondly, the panicle photosynthetic rate was still not saturated at very high air CO_2_ concentration (1800 μmol mol^−1^; Fig. 6a), which also differed from that of a typical leaf.

### Correlation between panicle photosynthetic gas exchange parameters and grain yield related agronomic traits

**Fig. 7:**
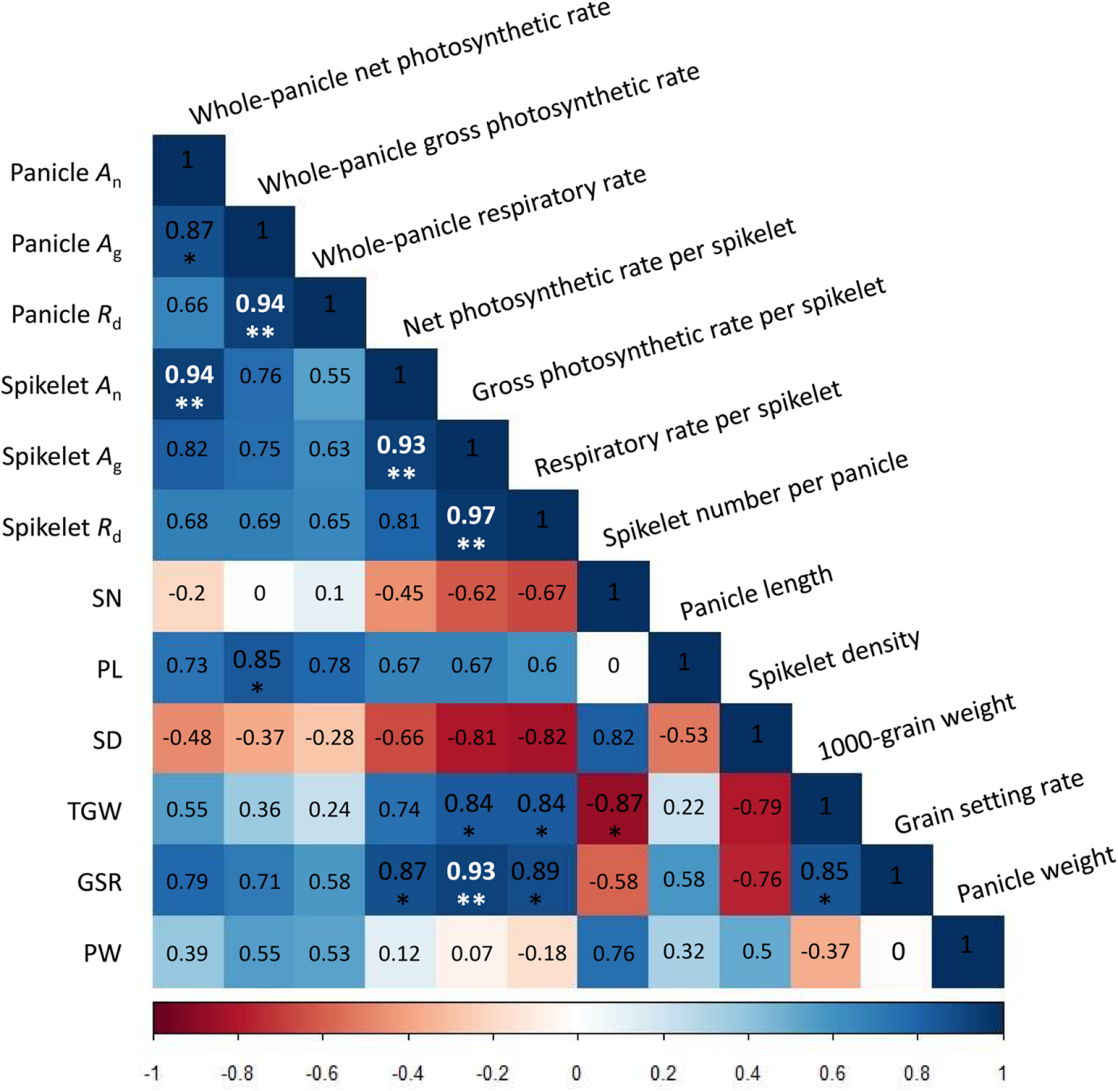
Correlation of panicle photosynthetic gas exchange parameters and grain yield related agronomic traits. Data were combined from 2015 and 2016 experiments shown in Table 1 and Table 4 (n = 12). Abbreviations are defined in the figure. The numbers in the figure are the Pearson correlation coefficients. *p-value < 0.001; **p-value < 0.0001

Finally, we studied the relationship between panicle photosynthetic gas-exchange parameters measured 5 days after heading (Table 1) and six grain yield related agronomic traits collected at harvest across 2 years, i.e., spikelet number per panicle, panicle length, spikelet density (defined as spikelet number per centimeter of panicle length), 1000-grain weight, grain setting rate and panicle weight (Table 4). Firstly, there were positive correlations between different panicle photosynthetic gas-exchange parameters (Fig. 7). For example, significant positive correlations were found between *A*_gross_ and *A*_net_ of a whole panicle (Pearson correlation coefficient r = 0.87, *p*-value < 0.001), between *A*_gross_ and *R*_d_ of a whole panicle (r = 0.94, *p*-value < 0.0001), between *A*_gross_ and *A*_net_ per spikelet (r = 0.93, *p*-value < 0.0001), and between *A*_gross_ and *R*_d_ per spikelet (r = 0.97, *p*-value < 0.0001). In contrast, both positive and negative correlations were found between grain yield related agronomic traits, e.g. significant negative correlations were found between 1000-grain weight and spikelet number per panicle, but significant positive correlations were found between 1000-grain weight and grain setting rate (Fig. 7). Notably, strong positive correlations were found between *A*_gross_, *A*_net_, *R*_d_ per spikelet and grain setting rate, 1000-grain weight. In particular, the Pearson correlation coefficient r is 0.93 (*p*-value < 0.0001) between *A*_gross_ per spikelet and grain setting rate (Fig. 7). Concurrently, we found that *A*_gross_, *A*_net_ and *R*_d_ of a whole panicle had a moderate positive correlation with panicle dry weight at harvest (r = 0.39 ~ 0.55); whereas *A*_gross_, *A*_net_ and *R*_d_ per spikelet were not correlated with panicle dry weight at harvest (r = –0.18 ~ 0.12). In addition, all the six panicle photosynthetic gas exchange parameters were found to be positively correlated with panicle length (r = 0.6 ~ 0.85), whereas they were negatively correlated with spikelet density (r = –0.82 ~ –0.28). Finally, no correlations were found between *A*_gross_, *A*_net_, *R*_d_ of a whole panicle and spikelet number per panicle (r = –0.2 ~ 0.1).

**Table 4:** Grain yield related agronomic traits of the rice cultivars at harvest

Year	Cultivar	Spikelet number	Panicle length (cm)	Spikelet density	1000-grain weight (g)	Grain setting rate	Panicle weight (g)
2015	YLY900	356 ± 24b	28.1 ± 1.5a	13.1 ± 1.4b	22.9 ± 0.7d	0.84 ± 0.07bc	7.4 ± 1b
	CY1000	444 ± 30a	21.8 ± 1.1c	21 ± 2.4a	21.6 ± 0.7d	0.8 ± 0.05c	8.7 ± 0.5a
	SY63	231 ± 21c	27.6 ± 0.9a	8.4 ± 0.6c	28.2 ± 1.3b	0.92 ± 0.04ab	6.4 ± 0.6bc
	9311	234 ± 40c	24.5 ± 1b	9.5 ± 1.3c	30.7 ± 0.4a	0.97 ± 0.02a	7.2 ± 1.1b
	XS134	253 ± 14c	17.1 ± 0.7d	14.8 ± 0.9b	25.2 ± 0.8c	0.74 ± 0.08c	5.7 ± 0.4c
2016	YLY900	359 ± 36b	32.7 ± 0.6a	10.9 ± 0.9d	22.2 ± 4c	0.83 ± 0.09bc	7 ± 0.9ab
	CY1000	410 ± 44a	26.5 ± 1c	13.8 ± 2.3bc	21.8 ± 0.5c	0.81 ± 0.03c	8.1 ± 0.5a
	SY63	216 ± 21c	30.5 ± 1.2b	6.7 ± 0.5e	29.6 ± 1.1ab	0.96 ± 0.01ab	6.4 ± 0.4b
	9311	211 ± 20c	27.2 ± 1.3c	8.2 ± 0.3e	30.8 ± 1.6a	0.96 ± 0.02a	6.3 ± 0.7bc
	XS134	222 ± 32c	18.2 ± 1.5e	11.2 ± 1.1 cd	24.1 ± 0.3bc	0.79 ± 0.05c	4.7 ± 0.3c
	YY538	420 ± 35a	21.6 ± 0.9d	17.2 ± 0.7a	20.5 ± 1.3c	0.77 ± 0.06c	6.9 ± 0.7ab
	YY17	403 ± 33a	23.9 ± 1.1d	15.5 ± 0.9ab	22.3 ± 1c	0.84 ± 0.08abc	7.4 ± 0.6ab

Data presented are mean values with s.d. (n = 5). Data followed by different letters are statistically different at *p*-value < 0.05

## Discussion

Reproductive organs of cereal crops have been shown photosynthetically active, and photosynthesis in ear of wheat and barley has attracted great research interest [12–15]. Current high-yield rice cultivars usually have much bigger ears with many spikelets compared with wheat or barley. For example, the newly bred rice cultivar YLY900, which have reached record-high yield of 15 tons ha^−1^ [36], and another even higher yielded rice cultivar CY1000, have an extra-large panicle size of exceeding 350 and 400 spikelets per panicle, respectively (Table 4). As a result, the green area of a rice panicle can even be larger than a flag leaf (Table 2). Furthermore, considering that the panicles locate at the upper part of a canopy, they can intercept a considerably amount of solar radiation. All these suggest a great role of panicle photosynthesis in rice grain filling. However, compared to leaf photosynthesis, panicle photosynthesis is much less studied, largely due to the methodological limitations [12, 22, 23]. Therefore, new tools and methods are needed to enable better characterization of rice panicle photosynthesis and provide an accurate evaluation of the impact of panicle photosynthesis on rice grain yield.

Here we reported the development of a new panicle photosynthesis P-chamber, which can be connected to a standard infrared gas analyzer to enable direct measurement of gas exchange parameters. Compared to previously reported cuvettes for rice or wheat ear gas exchange measurement [8, 17, 22, 37], the P-chamber has several new features. First, it is made air-tight with very limited leakage under different CO_2_ concentrations and flow rates, which enables accurate and fast measurement of gas exchange rate. Secondly, it has a large cuvette volume for gas exchange measurement of a whole rice panicle in situ. The large cuvette volume enables measurement of panicles of irregular shape. We expect that it can also be used to measure inflorescence of Arabidopsis, wheat, rape and soybean and other plant organs such as stem, fruit, and branch in situ. Moreover, the chamber is opaque and the PFD inside is uniformly distributed and can be precisely controlled, which enables studying response of photosynthesis to PFD.

Using the P-chamber, we studied quantitative characteristics of rice panicle photosynthesis. First of all, to facilitate comparison between tissues, gas exchange rates are normalized. There are three commonly adopted approaches to normalize gas exchange rates of foliar and non-foliar organs, i.e., (1) normalization based on organ number, which is widely used for cereal ears [25, 38]; (2) normalization based on area, which is commonly used for leaves [37, 39]; and (3) normalization based on chlorophyll content, which is applicable for both foliar and non-foliar organs [8, 40]. For ears of cereal crops, it is labor-saving and technically easier to measure gas exchange rate on a whole ear basis or on a spikelet basis rather than on an area basis or chlorophyll basis. However, what’s the relationship between gas exchange rates normalized on different bases? Here we show that gas exchange rate on a spikelet basis is highly correlated to gas exchange rate on an area basis (Pearson correlation coefficient r = 0.97−0.99; Fig. 3). This result indicates that we may use spikelet-based spike photosynthesis rate as a surrogate for the area-based spike photosynthesis rate, as the former one is non-intrusive and technically easier. For the seven rice cultivars used in this study, we have the following equation to convert sipkelet number to panicle area (Fig. 4):

1 $$ {\text{Panicle area}} = 0.266 * {\text{Spikelet number}} + 9.84 $$

Strikingly, *A*_net_ on a panicle basis is also highly correlated to *A*_net_ on an area basis for the seven rice cultivars used in this study (Pearson correlation coefficient r = 0.97; Fig. 3). This result indicates that panicle *A*_net_ is largely determined by *A*_net_ per unit area rather than its spikelet number or green surface area.

Secondly, we found that as a result of high respiratory rate and large panicle area, *A*_gross_ of a whole panicle 5 days after heading could be as high as 17 to 54 nmol s^−1^ under a PFD of 2000 μmol (photons) m^−2^ s^−1^. With an assumed typical photosynthetic rate of 25 μmol m^−2^ s^−1^ under a PFD of 2000 μmol (photons) m^−2^ s^−1^ [41–43], the *A*_gross_ of whole flag blades for the seven rice cultivars were 82 to 220 nmol s^−1^. This estimation resulted in the ratio between panicle *A*_gross_ and flag leaf *A*_gross_ to be 20−38% (Table 5). This result is comparable to a previous report, where *A*_gross_ of a panicle is 30% of a flag leaf [8]. Concurrently, though panicle photosynthetic activity gradually decreased during grain filling, it still maintained CO_2_ uptake activity even 40 days after heading, especially for the newly released super high yield hybrid rice cultivars, YLY900 and CY1000 (Fig. 5a). Maintaining photosynthetic activity of a panicle till the end of rice grain filling has been reported earlier as well [28, 40]. The contribution of panicle photosynthesis to rice grain yield cannot be precisely calculated in the current study due to the lack of the light environments for panicle throughout the grain filling season. However, the contribution of panicle photosynthesis to grain filling can be estimated based on measured rates of respiration. Specifically, if we assume a constant respiratory rate during the daytime, i.e., from 6:00 AM to 18:00 PM, the total daytime-respired CO_2_ during the whole grain filling season for the seven cultivars is 21–40 mmol (an integration of the Tot. *R*_d_ in Fig. 2), which is equivalent to 0.6-1.2 g CH_2_O and accounts for 12.1–17.0% of the panicle weight at harvest (Table 4). Namely, by refixation of daytime-respired CO_2_, panicle photosynthesis can apparently contribute 12–17% of the final grain yield. Considering that during most of the grain filling season, panicle maintains a positive carbon uptake (Fig. 5), we conclude that the apparent contribution of panicle photosynthesis to grain filling will be higher than 12%. Combination of the photosynthetic light response curve measured by P-chamber with an elaborate three-dimensional modeling of rice panicles and leaves [44], light environments within a canopy can be calculated and the daily and season-long photosynthetic contribution of a panicle can be precisely quantified.

**Table 5:** Estimated ratios of light saturated gross photosynthetic rates between a panicle and a flag leaf 5 days after heading in seven rice cultivars

Name	*A*_gross_ of a flag leaf (nmol s^−1^)	*A*_gross_ of a panicle (nmol s^−1^)	P/FL *A*_gross_
YLY900	185	48	0.26
CY1000	220	44	0.2
SY63	155	50	0.32
9311	141	54	0.38
XS134	82	17	0.21
YY538	122	26	0.21
YY17	181	36	0.2

Data measured in 2016 (n = 5). *A*_gross_ of a flag leaf, estimated light saturated gross photosynthetic rate of a whole flag leaf by assuming a constant light saturated photosynthetic rate of 25 μmol m^−2^ s^−1^ (the leaf area data was taken from Table 2); *A*_gross_ of a panicle, light saturated gross photosynthetic rate of a whole panicle (data was taken from Table 1); P/FL *A*_gross_, the ratio between light saturated gross photosynthetic rate of a panicle and that of a flag leaf

Thirdly, there are substantial inter-cultivar variations in panicle photosynthetic and respiratory rates 5 days after heading, both on a whole panicle basis and on a spikelet basis (Table 1). *Indica* cultivars have ~ 402% and ~ 693% higher panicle net photosynthetic rates per panicle and per spikelet than *japonica* and *japonica*-*indica* hybrid cultivars, respectively; meanwhile, *indica* cultivars have ~ 68% and ~ 83% higher panicle respiratory rates per panicle and per spikelet, respectively (Fig. 2). Overall, *indica* cultivars show ~ 94% and ~ 122% increase in panicle gross photosynthetic rates on the panicle basis and on the spikelet basis, respectively (Fig. 2). One of the factors contributing to these differences might be panicle morphology. For example, the high-yielding *japonica* rice cultivars usually have erect and densely-packed panicles [45], while the *indica* rice cultivars typically have drooping and loosely-packed panicles [46]. Indeed, highly negative correlations between spikelet density and respiratory rate per spikelet (Pearson correlation coefficient r = −0.82; Fig. 7), and between spikelet density and net photosynthetic rate per spikelet were found in this study (Pearson correlation coefficient r = −0.66; Fig. 7). The mechanisms by how spikelet density could influence both panicle photosynthesis and respiration are not clear. Inter-subspecies differences in photosynthetic apparatus and metabolism may also exist, which need to be further studied. Similar to this, there is a significant difference between leaf photosynthetic rates between *indica* and *japanica* cultivars, the molecular mechanism of which also awaits clarification [47, 48].

Fourthly, rice panicles show differences in photosynthetic response pattern to light intensities and CO_2_ concentrations compared to the leaves. Specifically, rice panicle has high photosynthetic light compensation point, and has high apparent CO_2_ compensation and saturation points. Consistent with our finding here in rice, wheat ear was also found to have higher CO_2_ compensation and saturation points than that of a flag leaf, which might be due to its high respiratory rate and different stomatal conductance response pattern [37].

Correlation study between panicle photosynthetic gas exchange parameters and grain yield related agronomic traits further shows a significant role of panicle photosynthesis in rice grain filling. Specifically, we found spikelet gross photosynthetic rates at early grain filling stage were strongly and significantly correlated with grain setting rate (r = 0.93) and 1000-grain weight (r = 0.84; Fig. 7). These results indicate that activity of the spikelet as a source and as a sink may closely linked. It is likely that the activity of the spikelet as a photosynthetic source promotes the activity of the grain as a sink. The detailed mechanism of this “source-sink” correlation is still not clear. Earlier studies on wheat offer a few possibilities: (1) panicle photosynthesis may supply oxygen to the hypoxic regions deep within the developing seed to increase its metabolic activity [49]; (2) the transpiration accompanying panicle photosynthesis not only supports the transfer of nutrients and signal molecules from xylem to spikelets, but also regulates panicle temperature to avoid over-heating [50, 51]; (3) panicle photosynthesis can generate assimilatory power, i.e., ATP and reduced ferredoxin, which are required to support glume nitrogen assimilation, a major source of nitrogen in wheat grain [52].

Finally, the method of directly measuring reproductive organ gas exchange rate has a range of potential applications. Firstly, it can help accurately simulate photosynthesis from non-foliar organs and evaluate their role in canopy photosynthesis based on three-dimensional canopy photosynthesis modeling [44, 53]. Secondly, as large natural variations of panicle photosynthetic and respiratory rates exist between cultivars, the genetic basis can be explored by measuring panicle gas exchange parameters in rice genetic populations. Thirdly, the diurnal and seasonal panicle respiratory rate change can be measured to trace grain filling dynamics to support identification of new options to improve panicle grain filling patterns [43, 54].

## Conclusions

Here we report the design and application of a new device, the P-chamber. The features of large cuvette volume, air tight, and programmable light intensity inside the P-chamber enable in situ characterization of photosynthetic gas exchange of a whole plant organ. With the P-chamber, for the first time, we characterized whole-panicle photosynthetic light and CO_2_ response patterns, quantified variations of panicle photosynthesis between cultivars and identified correlations between panicle photosynthetic gas exchange parameters and agronomic traits in rice. Further application of the P-chamber will facilitate study on photosynthetic characteristics and contribution of irregular photosynthetic organs, especially the non-foliar photosynthetic organs, e.g. inflorescence of cereals and other plant organs such as stem and branch.

## Materials and methods

### Plant materials and experimental design

Rice plants were grown in a paddy field at Songjiang breeding station of the Institutes of Plant Physiology and Ecology, Chinese Academy of Sciences, Shanghai, China (30°56’ 44” N, 121°8’ 1” E) in 2015 and 2016. Seeds were sown on seedbeds after germination on 1 June 2015 and 5 June 2016, and seedlings were transplanted to the field on 26 June 2015 and 29 June 2016. In both years, one plant was transplanted into a hill with a spacing of 20 cm between hills and 20 cm between rows. The cultivars were planted in plots. Plots were arranged in blocks with five replicates, each containing 56 (7 × 8) plants. Basal fertilizer was applied at a rate of 120 kg N ha^−1^, 65 kg P ha^−1^ and 65 kg K ha^−1^. Additional N fertilizer was top-dressed at a rate of 80 kg N ha^−1^ 3 weeks after transplanting. Weeds, pests and diseases were controlled following common agronomic practice in the region. In 2015, five rice cultivars were grown for experiments: two recently bred *indica* super high yield hybrid cultivars Y-Liang-You 900 (YLY900; http://www.ricedata.cn/variety/varis/614537.htm) and Chao-You 1000 (CY1000; http://www.ricedata.cn/variety/varis/616642.htm), one widely grown traditional *indica* hybrid cultivar Shan-You 63 (SY63; http://www.ricedata.cn/variety/varis/601174.htm), one *indica* inbred cultivar Yang-Dao 6 (9311; http://www.ricedata.cn/variety/varis/600611.htm) and one elite *japonica* inbred cultivar Xiu-Shui 134 (XS134; http://www.ricedata.cn/variety/varis/603176.htm). In 2016, in spite of the above mentioned 5 cultivars, two additional *japonica*-*indica* hybrid cultivars, Yong-You 538 (YY538; http://www.ricedata.cn/variety/varis/613563.htm) and Yong-You 17 (YY17; http://www.ricedata.cn/variety/varis/612347.htm), were grown for experiments. The heading date and harvest date of main stems for these rice cultivars in 2 years were given in Additional file 1: Table S1.

### Construction of the custom-designed panicle chamber

To enable direct and non-intrusive measurement of panicle photosynthesis, we built a panicle photosynthesis measurement chamber (P-chamber), which can be used together with infrared gas analyzers to measure photosynthetic and respiratory rates of a whole rice panicle. The chamber body is made of aluminum, with cooling fins and cooling fans on both sides (Fig. 1c). The chamber body has a dimension of 30 × 5 × 5 cm. The air inside the chamber was mixed with two air-mixing fans inside the chamber (F2008ES-05WAV, SHICOH IC FAN, Japan), light source and a PFD control system (Fig. 1c, d). The inner space is uniformly illuminated with 192 light-emitting diodes (LEDs; CREE XML T6, Guangdong Benbon Electrical Co., Ltd) on an 30 × 5 cm Aluminum substrate (Dongxing electronic technology Co., Ltd) for which the PFD can be precisely controlled to span from 0 to 2000 μmol (photons) m^−2^ s^−1^ (Fig. 1d). The PFDs in the P-chamber under different input voltages on LEDs were measured with an irradiance sensor (LightScout Light Sensor Reader, LightScout, Spectrum Technologies, Inc. USA). During measurement, the P-chamber is connected to a standard Infra-red gas analyzer Li-6400, and the rice panicle is held upright and fully enclosed in the chamber (Fig. 1a, b). Flow rate and CO_2_ concentration of influx air are controlled by LI-6400, and data are recorded and stored in LI-6400. Temperature inside the P-chamber was not controlled during measurement. The ambient air temperature for each measurement was recorded (see in Additional files 1: Table S2 and Table S3).

### Measurement of panicle photosynthetic gas exchange parameters

Twenty panicles were randomly selected and labeled at heading for each rice cultivar, which were used to measure photosynthetic gas exchange parameters and yield related agronomic traits. Three and five panicles among these twenty were used to measure whole-panicle net photosynthetic rate and dark respiratory rate in 2015 and 2016, respectively. During each measurement, the CO_2_ concentration was set to 400 μmol mol^−1^, the air flow rate was set to 700 μmol s^−1^. For net photosynthetic rate measurement, the PFD was set to 2000 μmol (photons) m^−2^ s^−1^; for dark respiratory rate measurement, the PFD was set to 0 μmol m^−2^ s^−1^. Each time when the gas exchange rate readings were stabilized, data were logged after matching the two infrared gas analyzers (IRGAs).

The rice cultivar YY17 was used for photosynthetic light and CO_2_ response curve measurements 10 days after heading in 2016. For photosynthetic light response curve measurement, the CO_2_ concentration was set to 400 μmol mol^−1^, the flow rate was set to 700 μmol s^−1^; and the light levels were manually changed in the following sequence: 2000, 1500, 1000, 700, 500, 300, 200, 100, 50, 25 and 0 μmol (photons) m^−2^ s^−1−^ during measurement. Panicles were maintained in the cuvette at each light level for about 4 min before photosynthetic rate was stabilized and recorded. For photosynthetic CO_2_ response curve measurement, the PFD was set to 2000 μmol (photons) m^−2^ s^−1^, the flow rate was set to 700 μmol s^−1^, and the reference CO_2_ concentration was set to 400 μmol mol^−1^. The panicles were maintained under the above described condition for 15 min. Then, the CO_2_ concentrations were manually changed in the following sequence: 400, 300, 150, 100 and 50 μmol mol^−1^ during measurement. Panicles were maintained in the cuvette at each CO_2_ concentration for about 4 min before photosynthetic rate was stabilized and recorded. Next, the CO_2_ concentration was set back to 400 μmol m^−2^ s^−1^ and maintained at that concentration for 15 min. Finally, the CO_2_ concentrations were manually changed in the following sequence: 600, 900,1200, 1500 and 1800 μmol mol^−1^. Again, panicles were maintained in the cuvette at each CO_2_ concentration for about 4 min before photosynthetic rate was stabilized and recorded.

### Calculation of panicle area and flag area

Areas of spikelets and branches in a panicle were calculated to obtain the total area of a panicle. Specifically, the non-degraded green spikelets were firstly detached from the panicle 5 days after heading; then, these spikelets were scanned with an Epson Perfection V300 Photo scanner (Epson, Tokyo, Japan; Additional file 1: Figure S1a). The areas of spikelets in the scanned images were obtained by image processing using ImageJ (US National Institutes of Health, Bethesda, USA). The flag leaf area was obtained in the same way (Additional file 1: Figure S1c).

The spikelet-less panicles were then spread out, scanned with an Epson Perfection V300 Photo scanner as well (Epson, Tokyo, Japan; Additional file 1: Figure S1b). The projected area of branches was obtained through image processing using ImageJ (US National Institutes of Health, Bethesda, USA). Branches were assumed as cylinders, and single-sided surface area of all branches on a panicle *S* was then calculated as:

2 $$ S\, = \,S_{bp} \, * \,\pi /2. $$

in which *s*_bp_ is the projected area of branches. Single-sided surface area of a whole panicle was calculated as a sum of the area of branches and the area of all spikelets.

### Agronomic traits measurement

Five of the twenty labeled panicles were sampled and stored in envelops individually for each cultivar at harvest. The panicle length was measured, and then the panicles were put in an oven for drying. The temperature of the oven was maintained at 110 °C for 1 h, and then switched to 70 °C and maintained for 3 days until samples were completely dried. Dry weight of each panicle was measured with an electronic balance AL104 (JB/T, METTLER TOLEDO, USA). The panicles were then threshed to determine the total spikelet number and filled grain number. The weight of all filled grains were measured to determine the 1000-grain weight.

### Data analysis

Statistical analyses were carried out using R-project (version 3.6.2). One-way analysis of variance (ANOVA) and the Scheffe post hoc test were used to compare data at a level of 5%. The Pearson correlation coefficients between panicle photosynthetic gas exchange parameters and grain yield related agronomic traits were calculated using the R package (Corrplot; version 0.84).

## Supplementary information

**Additional file 1: Figure S1.** Measurements for areas of spikelets, panicle branches and flag leaves 5 days after heading. **a** A scan photo of spikelets on a panicle. **b** A scan photo of panicle branches. **c** A scan photo of a flag leaf. **Table S1.** Heading date and harvest date of main stems for rice cultivars grown in 2015 and 2016. **Table S2.** Ambient air temperature (^o^C) during each panicle gas exchange measurement in 2015. **Table S3.** Ambient air temperature (^o^C) during each panicle gas exchange measurement in 2016.

## Data Availability

All data generated or analyzed during this study are included in the article and in Additional file 1.

## Abbreviations

9311: An *indica* inbred cultivar Yang-Dao 6
*A*_gross_ per unit area: Gross panicle photosynthetic rate on an area basis (μmol m^−2^ s^−1^)
*A*_net_ per unit area: Net panicle photosynthetic rate on an area basis (μmol m^−2^ s^−1^)
C_a_: Ambient air CO_2_ concentration (μmol mol^−1^)
CY1000: An *indica* super high yield hybrid rice cultivar Chao-You 1000
Panicle *A*_net_: Net photosynthetic rate of a whole panicle (nmol s^−1^)
Panicle *A*_gross_: Gross photosynthetic rate of a whole panicle (nmol s^−1^)
Panicle *R*_p_: Respiratory rate of a whole panicle (nmol s^−1^)
P-chamber: The custom-built chamber for panicle photosynthetic gas exchange rate measurements
PFD: Photon flux density (μmol m^−2^ s^−1^)
*R*_d_ per unit area: Panicle respiratory rate on an area basis (μmol m^−2^ s^−1^)
Spikelet *A*_net_: Net panicle photosynthetic rate on a spikelet basis (nmol s^−1^)
Spikelet *A*_gross_: Gross panicle photosynthetic rate on a spikelet basis (nmol s^−1^)
Spikelet *R*_d_: Panicle respiratory rate on a spikelet basis (nmol s^−1^)
SY 63: A traditional *indica* hybrid cultivar Shan-You 63
XS134: An elite *japonica* inbred rice cultivar Xiu-Shui 134
YLY900: An *indica* super high yield hybrid rice cultivar Y-Liang-You 900
YY17: A *japonica*-*indica* hybrid rice cultivar Yong-You 17
YY538: A *japonica*-*indica* hybrid rice cultivar Yong-You 538
