# Supplementary material for: An in situ approach to characterizing photosynthetic gas exchange of rice panicle
Source: Plant Methods. 2020 Jul 6;16:92. doi: 10.1186/s13007-020-00633-1 (PMC7336644; doi:10.1186/s13007-020-00633-1)
Supplement: Supplementary file 1 — Additional file 1: Figure S1. Measurements for areas of spikelets, panicle branches and flag leaves 5 days after heading. a A scan photo of spikelets on a panicle. b A scan photo of panicle branches. c A scan photo of a flag leaf. Table S1. Heading date and harvest date of main stems for rice cultivars grown in 2015 and 2016. Table S2. Ambient air temperature (oC) during each panicle gas exchange measurement in 2015. Table S3. Ambient air temperature (oC) during each panicle gas exchange measurement in 2016. [file 13007_2020_633_MOESM1_ESM.docx]

Additional file 1

**An *in situ* approach to characterizing photosynthetic gas exchange of rice panicle**

Tian-Gen Chang, Qing-Feng Song, Hong-Long Zhao, Shuoqi Chang, Changpeng Xin, Mingnan Qu, Xin-Guang Zhu^*^


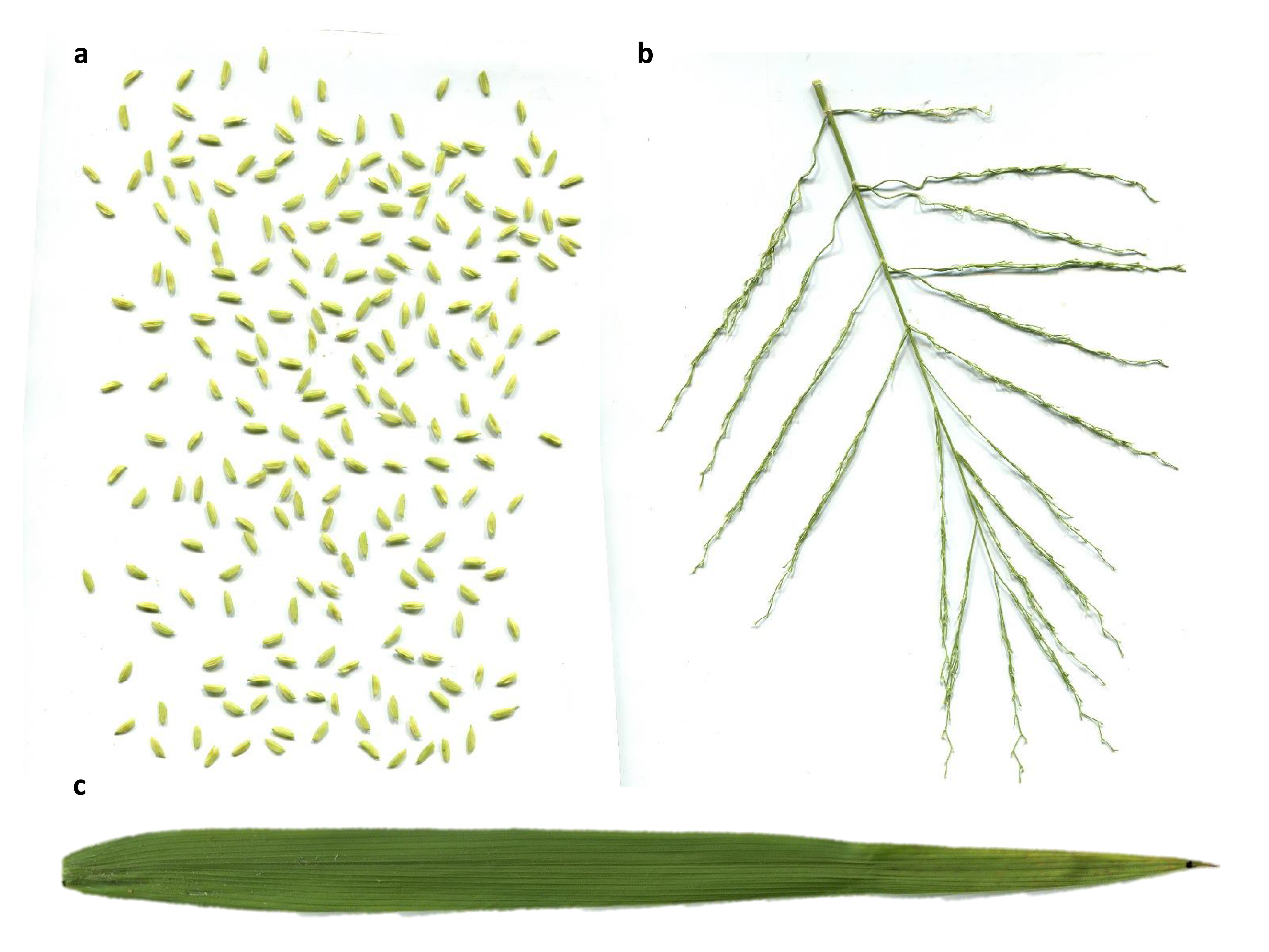


**Additional file: Figure S1.** Measurements for areas of spikelets, panicle branches and flag leaves 5 days after heading. **a**, A scan photo of spikelets on a panicle. **b**, A scan photo of panicle branches. **c**, A scan photo of a flag leaf.

**Additional file: Table S1.** Heading date and harvest date of main stems for rice cultivars grown in 2015 and 2016.

| **Name** | **Heading date (2015)** | **Harvest date (2015)** | **Heading date (2016)** | **Harvest date (2016)** |
| --- | --- | --- | --- | --- |
| YLY900 | 2 Sep. | 25 Oct. | 4 Sep. | 28 Oct. |
| CY1000 | 2 Sep. | 25 Oct. | 4 Sep. | 28 Oct. |
| SY63 | 27 Aug. | 25 Oct. | 29 Aug. | 28 Oct. |
| 9311 | 27 Aug. | 25 Oct. | 29 Aug. | 28 Oct. |
| XS134 | 6 Sep. | 5 Nov. | 7 Sep. | 8 Nov. |
| YY538 | - | - | 7 Sep. | 8 Nov. |
| YY17 | - | - | 15 Sep. | 8 Nov. |

**Additional file: Table S2.** Ambient air temperature (^o^C) during each panicle gas exchange measurement in 2015.

| Date | YLY900 | CY1000 | SY63 | 9311 | XS134 |
| --- | --- | --- | --- | --- | --- |
| 1 Sep. |  |  | 29.8-31.2 | 29.8-31.2 |  |
| 7 Sep. | 27.9-30.7 | 27.9-30.7 |  |  |  |
| 11 Sep. |  |  |  |  | 27.7-29.3 |

**Additional file: Table S3.** Ambient air temperature (^o^C) during each panicle gas exchange measurement in 2016.

| Date | YLY900 | CY1000 | SY63 | 9311 | XS134 | YY538 | YY17 |
| --- | --- | --- | --- | --- | --- | --- | --- |
| 3 Sep. |  |  | 34.8-35.5 | 34.8-35.5 |  |  |  |
| 9 Sep. | 32.9-34.0 | 32.9-34.0 |  | 32.9-34.0 |  |  |  |
| 12 Sep. |  |  | 29.8-30.9 |  | 29.8-30.9 | 29.8-30.9 |  |
| 20 Sep. |  |  |  |  |  |  | 27.7-28.6 |
| 21 Sep. | 28.0-28.6 | 28.0-28.6 |  |  |  |  |  |
| 22 Sep. |  |  |  |  | 27.8-29.1 | 27.8-29.1 |  |
| 24 Sep. |  |  | 28.0-29.0 | 28.0-29.0 |  |  | 28.0-29.0 |
| 6 Oct. | 28.6-32.6 |  |  |  | 28.6-32.6 |  |  |
| 7 Oct. |  | 28.4-29.3 |  | 28.4-29.3 |  |  |  |
| 8 Oct. |  |  |  |  |  |  | 28.3-29.2 |
| 9 Oct. |  |  | 24.5-28.9 |  |  | 24.5-28.9 |  |
| 17 Oct. | 26.6-33.2 | 26.6-33.2 |  |  |  |  |  |
| 18 Oct. |  |  |  |  | 25.9-32.9 | 25.9-32.9 | 25.9-32.9 |
| 1 Nov. |  |  |  |  | 18.3-24.3 | 18.3-24.3 | 18.3-24.3 |
